# A Novel Branched Al_2_O_3_/Silicon Rubber Composite with Improved Thermal Conductivity and Excellent Electrical Insulation Performance

**DOI:** 10.3390/nano11102654

**Published:** 2021-10-09

**Authors:** Yuge Ouyang, Xiaofei Li, Huafeng Tian, Liuyang Bai, Fangli Yuan

**Affiliations:** 1 College of Chemistry and Materials Engineering, Beijing Technology and Business University, Beijing 100048, China; tianhuafeng@th.btbu.edu.cn; 2 State Key Laboratory of Multiphase Complex Systems, Institute of Process Engineering, Chinese Academy of Sciences (CAS), Beijing 100190, China; xiaofeili@ipe.ac.cn (X.L.); flyuan@ipe.ac.cn (F.Y.); 3 College of Energy Engineering, Huanghuai University, Zhumadian 463000, China

**Keywords:** polymer composites, thermal conductivity, Al_2_O_3_, continuous network, electrical insulation

## Abstract

In this paper, we report a thermal conductive polymer composite that consists of silicone rubber (SR) and branched Al_2_O_3_ (B-Al_2_O_3_). Owing to the unique two-dimensional branched structure, B-Al_2_O_3_ particles form a continuous three-dimensional network structure by overlapping each other in the matrix, serving as a continuous heat conductive pathway. As a result, the polymer composite with a 70 wt% filler achieves a maximum thermal conductivity of 1.242 Wm^−1^ K^−1^, which is equivalent to a significant enhancement of 521% compared to that of a pure matrix. In addition, the composite maintains a high volume resistivity of 7.94 × 10^14^ Ω·cm with the loading of 70 wt%, indicating that it meets the requirements in the field of electrical insulation. Moreover, B-Al_2_O_3_ fillers are well dispersed (no large agglomerates) and form a strong interfacial adhesion with the matrix. Therefore, the thermal decomposition temperature, residual mass, tensile strength, modulus and modulus of toughness of composites are significantly improved simultaneously. This strategy provides new insights for the design of high-performance polymer composites with potential application in advanced thermal management in modern electronics.

## 1. Introduction

With the advent of the 5G era, electronic devices and equipment are developing in the direction of miniaturization and integration [1,2,3]. Therefore, the large amount of heat generated during the high-speed operation of electronic equipment places higher requirements on the thermal diffusion performance of polymer materials. However, due to the high disorder of molecular segments, polymers possess poor thermal conductivity (generally not higher than 0.2 Wm^−1^ K^−1^), which limits the wider application of polymer materials in the field of electronic packaging [4,5,6]. In recent years, the introduction of fillers with high thermal conductivity into polymer matrix to prepare polymer composites has attracted a considerable amount of attention, and it has been confirmed that this is an effective way to enhance the thermal conductive property of polymer matrices in academia and industry [7,8]. Traditional thermal conductive fillers used to prepare high thermal conductivity polymer composites include metal fillers (Al and Cu), carbon-based materials (graphene and carbon nanotubes) and ceramic fillers (AlN, BN, Al_2_O_3_ and SiC) [9,10,11]. Unfortunately, the addition of metal fillers or carbon-based fillers tends to damage the insulation property of the material to a certain extent, while ceramic fillers show unquestionable advantages due to their high thermal conductivity and electrical resistivity [12]. Although nitride fillers possess a higher thermal conductivity than oxide fillers, their expensive prices are not suitable for practical applications, while Al_2_O_3_ has been widely studied because of its high cost performance and rich natural resources [13,14]. However, adding Al_2_O_3_ to achieve high thermal conductivity generally requires a high loading [15,16], which will inevitably cause the following problems: firstly, more filler–matrix interfaces are formed in the composite system, leading to high interfacial thermal resistance due to strong phonon scattering and lower overall thermal conductivity of the material [17]; secondly, the excessive introduction of the second phase produces a large number of defects in the matrix, resulting in an increase in system viscosity and a decrease in mechanical properties [18,19].

In order to solve the above problems, the research mainly focused on the following three aspects: Firstly, researchers improved the interfacial adhesion between the filler and matrix by modifying the surface of Al_2_O_3_ particles to form a chemical or hydrogen bond with the matrix, which optimized the two-phase interface and weakened the interfacial thermal resistance [14,20,21,22,23]. Zhang and coworkers improved the interfacial adhesion between Al_2_O_3_ and the matrix by modifying the Al_2_O_3_ filler with acrylate grafted siloxane copolymers, which not only increased the thermal conductivity of SR by nearly 10 times but also greatly reinforced the tensile properties of the matrix [18]. Secondly, Al_2_O_3_ fillers were hybridized with other types of filler containing various morphologies or particle sizes to construct a continuous heat conduction network in the matrix using the “bridge” effect between particles [24,25,26,27]. For example, a small number of Ag nanoparticles were mixed with Al_2_O_3_ plates and distributed among them to form a continuous heat conduction network. This reduced the interfacial thermal resistance between Al_2_O_3_ fillers during heat transfer and improved the thermal conductivity of epoxy resin to about 6.71 Wm^−1^K^−1^. Importantly, the composites still maintained excellent mechanical and electrical insulation properties [28]. In addition to 0D Ag particles, Al_2_O_3_ fillers were also hybridized with 1D carbon fiber, carbon nanotubes, 2D BN or graphene to build a continuous heat conduction network [25,29,30,31,32,33]. Thirdly, various preparation processes, such as high-temperature sintering and vacuum filtration, were employed to construct a continuous Al_2_O_3_ filler network structure in the matrix [34,35,36]. Tian et al. prepared a thermally conductive epoxy/Al_2_O_3_ composite with an enhanced thermal conductivity of 2.58 Wm^−1^ K^−1^ by constructing an Al_2_O_3_ framework via a simple protein foaming process, followed by sintering at 1550 °C [37]. Yu et al. built an effective thermal transport highway with a vacuum-assisted infiltration method to promote contact among Al_2_O_3_ fillers to form bridges in the matrix [38,39]. It can be seen that various strategies were carried out to decrease the interfacial thermal resistance between Al_2_O_3_ fillers and a polymer matrix in order to increase the thermal conductivity of composites. However, it is still a challenge to prepare thermally conductive and electrical insulating composites with a simple and feasible method in order to realize the continuous distribution of Al_2_O_3_ in a matrix and maintain the excellent mechanical and electrical insulation properties of the materials.

In this work, branched Al_2_O_3_ (B-Al_2_O_3_) was used to prepare high thermal conductive and electrical insulated silicone rubber (SR) composites. B-Al_2_O_3_ fillers were well dispersed in the matrix, and it was easier to form a continuous heat conduction network by overlapping them each other, which reduced the interfacial thermal resistance during heat transfer. When the loading was 70 wt%, the thermal conductivity of the composite reached 1.242 Wm^−1^ K^−1^, which was 521% higher than that of pure SR. Importantly, due to the high resistivity of Al_2_O_3_ material, the composites maintained excellent electrical insulation properties. In addition, B-Al_2_O_3_ particles formed a strong interfacial adhesion with the matrix; thus, the thermal decomposition temperature, residual mass, tensile strength, modulus and modulus of toughness of the composites were greatly improved. These experimental results show that B-Al_2_O_3_ is a promising filler for the preparation of SR composites with high thermal conductivity and high insulation.

## 2. Materials and Methods

### 2.1. Materials

The B-Al_2_O_3_ fillers were prepared by a heat treatment of nanospherical Al_2_O_3_ at 1250 °C for 4.5 h. The preparation method and particle characteristics of spherical Al_2_O_3_ were presented in our previous papers [40]. The SR matrix (polydimethylsiloxane) was purchased from Wacker Chemical (China) Co., Ltd. (Shanghai, China). The crosslinking agent methyltris (methylethylketoximino) silane and catalyst dibutyltin dilaurate were provided by Macklin (Shanghai, China).

### 2.2. Preparation of B-Al_2_O_3_/SR Composites

The preparation process of B-Al_2_O_3_/SR composites with different loading was as follows: a certain amount of B-Al_2_O_3_ was added into the SR matrix; the powder and the matrix were preliminarily mixed by an electric mixer; and then the mixture was further dispersed by a three-roll grinder with high shear force. A certain type of crosslinking agent and catalyst were added to the mixture, which was then mixed again using an electric dispersant. Next, the dispersed mixture was degassed in a vacuum drying oven for 30 min and then poured into the polytetrafluoroethylene mold for a further curing reaction. After the samples were cured at room temperature for 24 h, they were taken out of the mold and placed in a drying oven with a constant temperature of 80 °C for 12 h to obtain the B-Al_2_O_3_/SR composite.

### 2.3. Characterization

The detailed morphologies of prepared B-Al_2_O_3_ particles and fracture surfaces of B-Al_2_O_3_/SR composites were observed using a field emission scanning electron microscope (FESEM, JSM-6700F, Tokyo, Japan) equipped with an energy dispersive X-ray spectrometer (EDX). Microscopic photographs with three different magnifications were used for particle size measurement of B-Al_2_O_3_ particles, and a total of 1000 particles were measured to obtain particle size distribution. Thermal conductivities of composites were measured using a thermal constant analyzer (TPS2500S, Hot Disk, Göteborg, Sweden), and samples were at a thickness of about 1 mm. A thermogravimetric analyzer (TG-DTA 6300 Instrument, SII NanoTechnology Inc., Tokyo, Japan) was applied to characterize the weightlessness behavior of B-Al_2_O_3_/SR composites in nitrogen with a heating rate of 10 °C over a temperature range from room temperature to 1000 °C. The volume resistivity of samples was analyzed based on a Keithley Model 6517A electrometer with an 8009 Resistivity Test Fixture equipped with ring electrodes. An Agilent 4294A impedance analyzer was employed to test the dielectric constant and dielectric loss of composites over a wide frequency range from 100 Hz to 1 MHz at room temperature. Specimens with dumbbell shapes were examined using a universal testing machine (AI-7000M, GOTECH, Taiwan, China) at the crosshead speed of 2 mm/min to obtain the tensile properties of SR composites.

## 3. Results and Discussion

### 3.1. Characterizations of B-Al_2_O_3_ Particles

**Figure 1:**
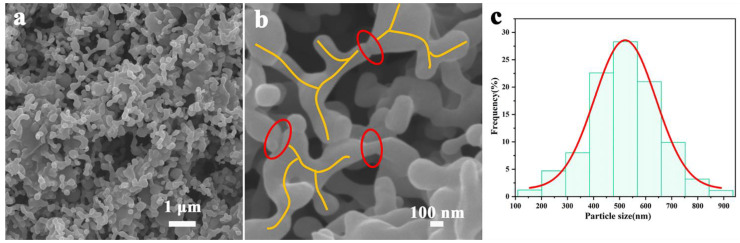
(**a**,**b**) SEM images and (**c**) particle size distribution of B-Al_2_O_3_.

The detailed characterizations of prepared B-Al_2_O_3_ particles by high-temperature sintering of Al_2_O_3_ nanospheres are presented in Figure 1. We can see from Figure 1a that the particles display irregular branched structures, which are due to the mass transfer between spherical particles during high-temperature sintering and, thus, form a more continuous structure compared with 0D spheres. Meanwhile, the 2D B-Al_2_O_3_ particles show good dispersion (no agglomerated particles are observed), which is very conducive to uniform dispersion in the matrix to form a more continuous heat conduction network. A more enlarged branched structure can be seen in Figure 1b. As marked by the yellow line, each particle possesses more than three branch structures, which promote the overlapping distribution of the fillers. The structures marked by the red ellipse in Figure 1b are the sintering necks formed by the sintering mass transfer of raw spherical particles; the formation of necks significantly reduces the number of passed interfaces of heat and decreases the direct interfacial thermal resistance between the filler and matrix. After high-temperature heat treatment, the defect concentration in the particles is reduced, which weakens the phonon scattering to a certain extent [17,41]. The smooth surfaces of particles (no bulge or depression is observed) is very beneficial for reducing the viscosity of the system to improve the processability of composites. Figure 1c shows the size distribution of branched structure of B-Al_2_O_3_, and the length of the branched structure is distributed from 100 to 900 nm (mainly about 500 nm). Therefore, we speculate that the unique structure tends to form a more continuous structure in the matrix at a lower loading than the common irregular fillers.

### 3.2. Microstructure of B-Al_2_O_3_/SR Composites

**Figure 2:**
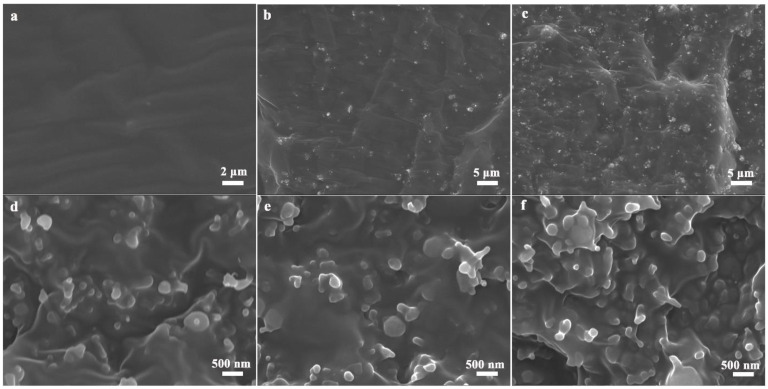
SEM images of fracture surface of (**a**) neat SR and B-Al_2_O_3_/SR composites with different filler content of (**b**) 10 wt%, (**c**) 30 wt%, (**d**) 50 wt%, (**e**) 60 wt% and (**f**) 70 wt%.

The cross-section morphologies of the composites with various loadings are shown in Figure 2. It can be seen from Figure 2a that the pure SR material maintains a smooth fracture surface, and no impurities are observed in the material. Figure 2b,c are SR composites with loadings of 10 wt% and 30 wt% B-Al_2_O_3_, respectively, and the particles are evenly distributed in the matrix (no clusters). As the fillers are far away from each other, the thick SR layer between them hinders the formation of a continuous filler network. Therefore, it is difficult to form a continuous three-dimensional heat transfer network in the system. As the loading continues to increase, the fillers gradually begin to contact (Figure 2d,e); thus, the overlapping of the branched structures of fillers promotes the formation of a continuous heat conduction network in the matrix. What is important is that the particles are still evenly dispersed in the matrix even as the loading reaches 70 wt% (Figure 2f), which is closely related to the surface smoothness of B-Al_2_O_3_ particles and is key to the formation of efficient heat transfer pathways. In addition, it can be clearly observed that there is no obvious gap between the filler and the matrix, indicating that the interface adhesion between the two is good, which not only helps to reduce the interfacial thermal resistance between the filler and the matrix but is also crucial to improving the comprehensive properties (mechanical properties and thermal properties) of the composites.

**Figure 3:**
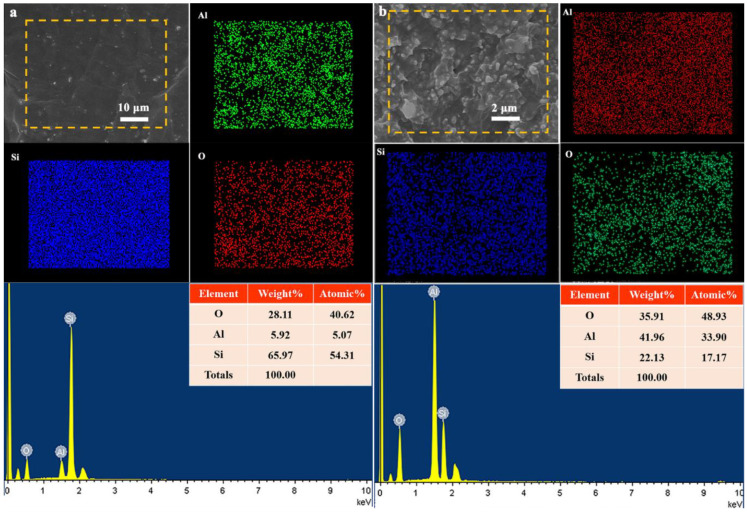
EDS scanning spectral results of the elements O, Al and Si: (**a**) 10 wt% B-Al_2_O_3_/SR composites and (**b**) 70 wt% B-Al_2_O_3_/SR composites.

The cross-sectional element distribution of the composite is analyzed by EDS (Figure 3). The uniform and continuous distribution of the Al element indicates that the B-Al_2_O_3_ filler is uniformly distributed in the matrix (even at high loading). The results further demonstrate that the Al_2_O_3_ and the SR matrix are mixed more uniformly, and there is no agglomeration of particles caused by high loading.

### 3.3. Thermal Conductive Property of B-Al_2_O_3_/SR Composites

**Figure 4:**
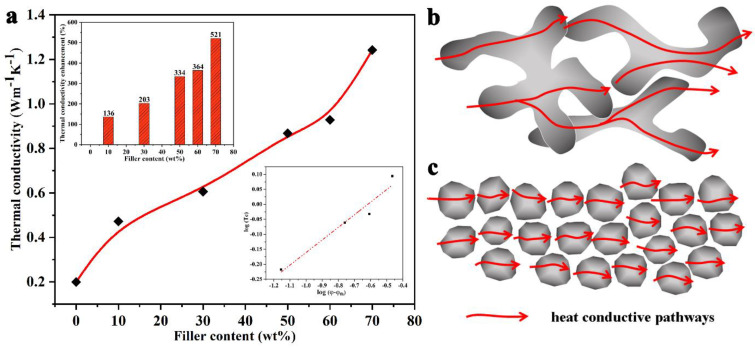
(**a**) Thermal conductivity of the SR composites filled with different contents of B-Al_2_O_3_ and heat conductive pathways of composites with (**b**) B-Al_2_O_3_ and (**c**) common irregular fillers.

The thermal conductivity of SR composites with various loadings of B-Al_2_O_3_ is shown in Figure 4. As presented in Figure 4a, pure SR exhibits poor thermal conductivity of 0.2 Wm^−1^ K^−1^, which is very close to the value reported in the literature [42,43]. With the addition of B-Al_2_O_3_, the thermal conductivity of the composites increases monotonously, and the increasing rate shows a rapid trend at first, which slows down slightly and then increases rapidly. For example, the thermal conductivity of the composite reaches 0.472 Wm^−1^ K^−1^ at the loading of 10 wt%, which is 136% higher than that of pure SR, suggesting the superiority of B-Al_2_O_3_ in enhancing the thermal conductivity of polymers. When the particle loading of B-Al_2_O_3_ increases from 30 wt% to 50 wt%, the thermal conductivity of the SR composite increases from 0.606 Wm^−1^ K^−^^1^ to 0.868 Wm^−1^ K^−1^. The increasing rate of thermal conductivity at this stage is relatively slow compared with the rate increased by adding 10 wt% B-Al_2_O_3_. In the mixed system, increasing the filler loading creates more heat transfer channels and introduces more filler–matrix interfaces. The numbers of channels and interfaces are two competitive factors, which jointly determine the final thermal conductivity of the material. Therefore, we speculate that the increase in the number of interfaces slows down the increasing rate of thermal conductivity at this stage. With the continuous addition of B-Al_2_O_3_, the increase in heat transfer pathways plays a leading role in improving the overall thermal conductivity of the material, and the thermal conductivity of the material reaches 0.928 Wm^−1^ K^−1^ and 1.242 Wm^−1^ K^−1^, respectively, while the loadings are 60 wt% and 70 wt%, which are 364% and 521% higher than that of pure SR, respectively. Moreover, the composites show no saturation effect for the thermal conductivity as a function of the filler loading fraction. The saturation effect is attributed to a tradeoff between the enhancement in thermal conductivity as more fillers are added and the decrease in the thermal conductance as the thermal interface resistance between the filler−filler and filler−matrix interfaces increases. The lower right inset in Figure 4a shows the experimental results and theoretical fitting in a log−log scale, and one can predict that the thermal conductivity of the composites will continue to improve with the addition of B-Al_2_O_3_ [44]. Compared with common irregular filler particles, the advantage of 2D B-Al_2_O_3_ in improving the thermal conductivity of the composite can be explained by Figure 4b,c. By overlapping the branched structures, it is easier to build a continuous and fast channel for the diffusion of heat (thermal percolation threshold can be reached at a lower loading) [45], which reduces the number of interfaces that heat must pass through, thereby weakening the influence of the interface and reducing the interfacial thermal resistance. Avoiding heat passing through the two-phase interface (through the polymer layer) is the most effective and direct way to improve the thermal conductivity of composites.

### 3.4. Thermal Stability of B-Al_2_O_3_/SR Composites

**Figure 5:**
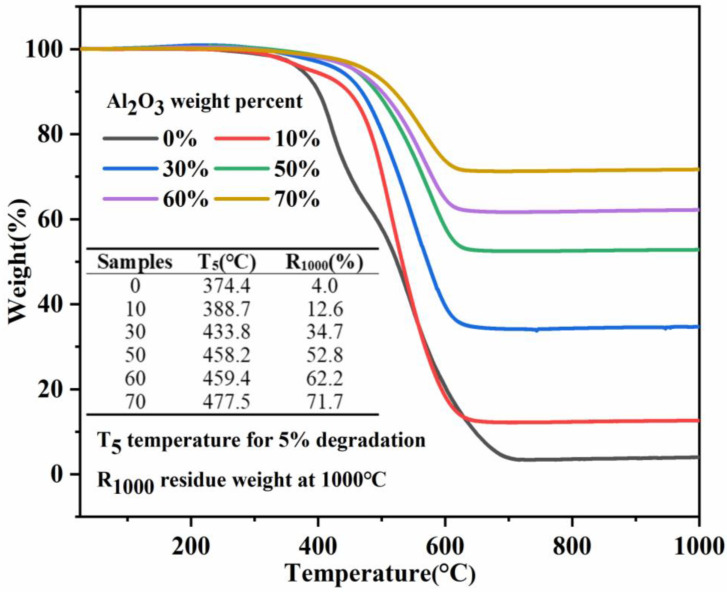
The weight loss curves of B-Al_2_O_3_/SR composites.

Thermal stability is an important factor for SR materials used in the field of electrical insulation. The thermal weight loss behavior of pure SR and its composites in a nitrogen atmosphere were characterized, as shown in Figure 5. The temperature at 5% weight loss is defined as the initial decomposition temperature, and the mass at 1000 °C is the residual mass. These two parameter values are also shown in Figure 5. The initial decomposition of pure SR occurs at about 374.4 °C. With the addition of B-Al_2_O_3_, the initial decomposition temperatures of SR composites increase continuously. For instance, the initial decomposition temperatures of composites with a load of 50 wt% and 70 wt% are 458.2 °C and 477.5 °C, respectively, which increase by 83.8 °C and 103.1 °C, respectively, compared with pure materials. Moreover, the addition of the filler also significantly improves the residual quality of the composite at 1000 °C. The improvement in these two parameters shows that the composites have a stronger resistance to thermal decomposition, which is mainly due to the excellent interfacial adhesion between B-Al_2_O_3_ and the matrix and the uniform dispersion of particles, which hinders the movement of molecular chain segments during heating and delays the decomposition of the material [46].

### 3.5. Electrical Properties of B-Al_2_O_3_/SR Composites

**Figure 6:**
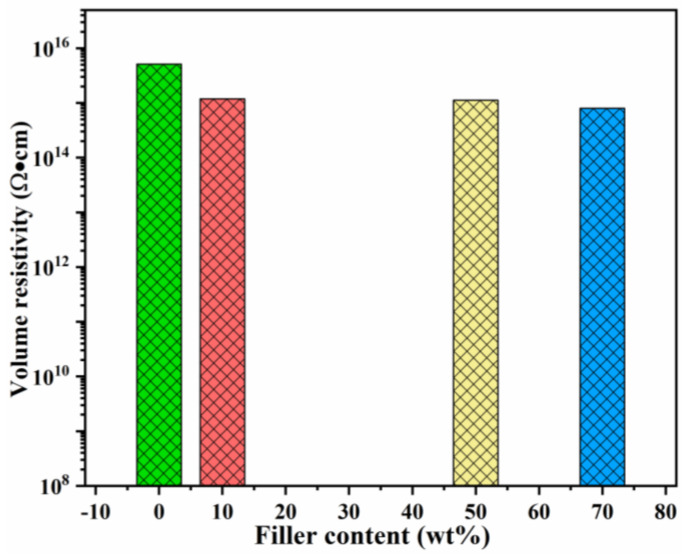
Volume resistivity of the B-Al_2_O_3_/SR composites.

Volume resistivity is used to characterize the electrical conductivity of a material and is an important indicator of its insulation performance. The main factors affecting the resistivity of materials are carrier type, carrier number and carrier mobility. The volume resistivities of SR and its composites are shown in Figure 6. The values of volume resistivity of the composites decrease gradually with the addition of B-Al_2_O_3,_ and the resistivity of the composites with filler loading of 10 wt%, 50 wt% and 70 wt% are 1.18×10^15^ Ω·cm, 1.12 × 10^15^ Ω·cm and 7.94 × 10^14^ Ω·cm, respectively. The introduction of the second phase forms an interface region in the matrix. The increasing filler leads to the overlap of the interface region and the formation of conductive paths, which increases the carrier mobility and decreases the volume resistivity. Even at the maximum loading of 70 wt%, the volume resistivity of the composite is five orders of magnitude higher than the critical value of electrical insulation (1 × 10^9^ Ω·cm) [28], indicating that the prepared B-Al_2_O_3_/SR composites still possess excellent insulation properties and meet the application in the field of electronic packaging.

**Figure 7:**
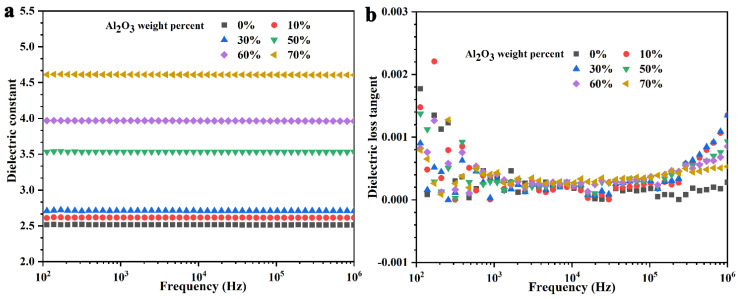
(**a**) Dielectric constant and (**b**) dielectric loss of the neat SR and B-Al_2_O_3_/SR composites.

A dielectric constant characterizes the polarization degree of materials under the action of an electric field. Different application fields require various dielectric constants. When used as a capacitor, the dielectric constant of the material needs to be larger, while a smaller dielectric constant is needed in an insulating medium. The value of a dielectric constant of composite is determined by three aspects: the polymer matrix, particles and the interface region formed by them. The change of a dielectric constant of SR and its composites with frequency is shown in Figure 7a. In the whole test frequency range, the dielectric constant of pure SR is basically unchanged with the change in frequency (about 2.51), which is due to the siloxane used as a non-polar polymer and the fact that there are only electron and ion polarizations caused by impurity ions in the high frequency region [47]. With the decrease in frequency, no new polarization appears, and the dielectric constant remains unchanged. With the increase in B-Al_2_O_3_ content, the dielectric constant of the composite increases gradually, but it remains stable in the whole tested frequency range. For example, for the composites with the addition of 50 wt% and 70 wt%, the dielectric constants are basically stable at about 3.53 and 4.61, respectively. The increasing dielectric constant of the composites is attributed to the fact that the inherent dielectric constant of Al_2_O_3_ (9.8 at 1 MHz) is larger than the SR [48]. However, the dielectric constant of the composites does not notably increase, which can be attributed to the strong interface between the particle surface and the matrix hindering the dipole polarization [49]. Figure 7b shows the change in the dielectric loss of SR and its composites with frequency. Electric conductive loss caused by electrical conduction and polarization loss caused by relaxation polarization will be produced in dielectric materials under the action of an external electric field. SR material has poor conductivity and is a non-polar molecule; thus, its electric conductive loss and polarization loss are low, and the dielectric loss of pure SR is very low in the whole frequency range. With the addition of B-Al_2_O_3_, the dielectric loss of the composite barely increases, which also shows that the added B-Al_2_O_3_ has high purity and almost no impurity molecules or water that increase the dielectric loss.

### 3.6. Tensile Properties of B-Al_2_O_3_/SR Composites

**Figure 8:**
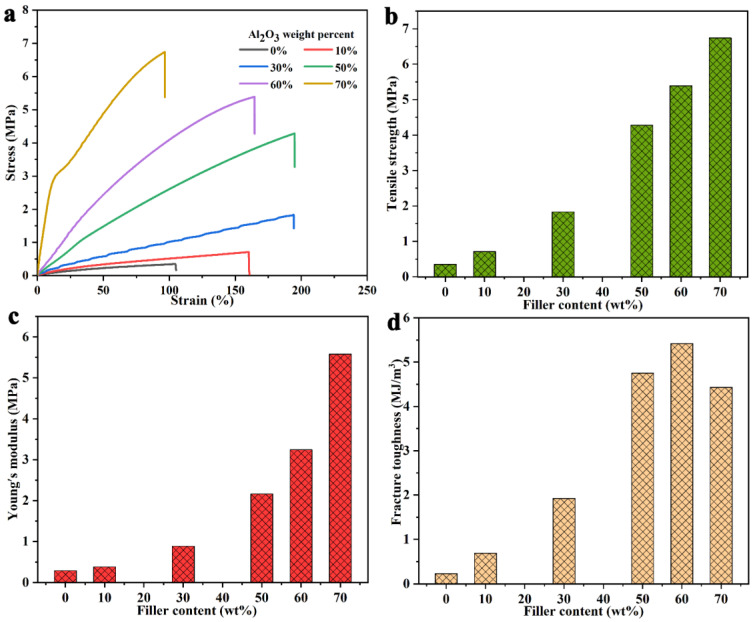
(**a**) Stress–strain curves, (**b**) strength, (**c**) modulus and (**d**) modulus of toughness of neat SR and Al_2_O_3_/SR composites.

We also evaluated the tensile properties of SR and its composites. The tested stress–strain curve (Figure 8a), tensile strength (Figure 8b), Young’s modulus (Figure 8c) and modulus of toughness (Figure 8d) are shown in Figure 8. The tensile strength and Young’s modulus of pure SR material are low, at 0.35 MPa and 0.29 MPa, respectively, and its elongation at the break is 104%. With the addition of B-Al_2_O_3_, the tensile strength and Young’s modulus of the composites gradually increase. For example, the tensile strength and Young’s modulus of the composites with a loading of 50 wt% are 4.28 MPa and 2.16 MPa, respectively, which are about 1123% and 645% higher than those of pure SR, respectively. For composites with a 70 wt% filler, the tensile strength and Young’s modulus are 6.74 MPa and 5.58 MPa, respectively, which increase by 1826% and 1824% compared with those of pure SR, respectively. The improvements in these two parameters show that B-Al_2_O_3_ improves the strength of SR material, which should be attributed to the good dispersion of B-Al_2_O_3_ in the matrix and the formation of strong interfacial adhesion with the matrix [50,51]. In addition, it can be seen from Figure 8a that when the B-Al_2_O_3_ loading increases from 10 wt% to 50 wt%, the elongation at the break of the composites gradually increases, and the elongation at the break of the composite with 50 wt% B-Al_2_O_3_/SR is 195%. With the continuous addition of B-Al_2_O_3_, the elongation at break decreases, and the elongation at break of 70 wt% B-Al_2_O_3_/SR is about 96%, lower than that of pure SR. This is because Al_2_O_3_ itself is a rigid material, reducing the flexibility of the SR composites. The modulus of toughness calculated by measuring the area under the stress–strain curve of the materials is shown in Figure 8d [52]. When the loading is 60 wt%, the maximum modulus of toughness of the material is 5.42 MJ/m^3^, which increases by 2257% relative to that of pure SR (0.23 MJ/m^3^). When it continues to increase to 70 wt%, the modulus of toughness decreases slightly, but it is still much higher than that of pure SR. The gradual improvements in tensile strength, Young’s modulus and modulus of toughness of SR composites show that the B-Al_2_O_3_ used in this work is a very promising filler to prepare high-strength and high thermal conductivity composites.

## 4. Conclusions

In summary, the B-Al_2_O_3_ particles were used to reinforce the thermal conductive performance of the SR matrix. Because the branched structures were overlapped with each other, a continuous heat transfer network was formed in the matrix, which reduced both the number of interfaces that heat must past though and the interfacial thermal resistance. The maximum thermal conductivity of the SR composite was 1.242 Wm^−1^ K^−1^, which is 521% higher than that of pure SR. At the same time, the composites demonstrated excellent electrical insulation performances, and the volume resistivity was 7.94 × 10^14^ Ω·cm when the loading was 70 wt%. This was still far beyond the critical resistance for electrical insulation (10^9^ Ω·cm), meeting the requirements for electrical insulation performance. Even with high loading, B-Al_2_O_3_ had good dispersion in the matrix and formed a strong interfacial adhesion with the SR. The initial decomposition temperature and residual mass of composites were greatly improved. The tensile test results showed that the tensile strength, elastic modulus and modulus of toughness of the composites were dramatically improved, indicating that the composites had a stronger ability to resist external stress. Therefore, this study provides a new perspective for the design and preparation of high-performance thermal conductive and insulating composites.

## Data Availability

The data presented in this study are available on request from the corresponding author.
